# Epidemiological variations and trends in glaucoma burden in the Belt and Road countries

**DOI:** 10.1186/s12886-024-03464-z

**Published:** 2024-04-25

**Authors:** Xiangwu Chen, Yingxi Zhao, Anjing Zhang, Yanping Zhou, Min Li, Xuelin Cheng, Yajun Zhao, Shijia Yang, Zhaoyu Zhang, Xiaopan Li

**Affiliations:** 1 Department of Ophthalmology, Zhongshan Hospital, Shanghai Medical College of Fudan University, Shanghai, 200032 China; 2 Department of Ophthalmology, Eye Hospital of Wenzhou Medical University, Wenzhou, 310020 Zhejiang Province China; 3 Department of Rehabilitation Medicine, Huashan Hospital, Shanghai Medical College of Fudan University, Shanghai, 200040 China; 4 Department of Neurorehabilitation Medicine, First Rehabilitation Hospital of Shanghai, Kongjiang Branch, theYangpu District, Shanghai, 200092 China; 5 Department of Health Management Center, Zhongshan Hospital, Shanghai Medical College of Fudan University, 180 Fenlin Rd., Xuhui, Shanghai, China; 6 Department of General Practice, Zhongshan Hospital, Shanghai Medical College of Fudan University, Shanghai, 200032 China

**Keywords:** B&R countries, Glaucoma, Burden of disease, Years lived with disability (YLDs), Trend analysis, Average annual percent change

## Abstract

**Background:**

Analyzing the glaucoma burden in "Belt and Road" (B&R) countries based on age, gender, and risk factors from 1990 to 2019 in order to provide evidence for future prevention strategies.

**Methods:**

We applied global burden of disease(GBD) 2019 to compare glaucoma prevalence and Years lived with disabilities (YLDs) from 1990 to 2019 in the B&R countries. Trends of disease burden between 1990 and 2019 were evaluated using the average annual percent change and the 95% uncertainty interval (UI) were reported.

**Results:**

From 1990 to 2019, most B&R countries showed a downward trend in age-standardized prevalence and YLDs (all *P* < 0.05). Additionally, only the age-standardized YLDs in males of Pakistan has a 0.35% increase (95%CI:0.19,0.50,*P* < 0.001), and most B&R countries has a decline(all *P* < 0.05) in age-standardized YLDs in every 5 years age group after 45 years old except for Pakistan(45–79 years and > 85 years), Malaysia(75–84 years), Brunei Darussalam(45–49 years), Afghanistan(70–79 years). Finally, in all Central Asian countries, the age-standardized YLDs due to glaucoma caused by fasting hyperglycemia demonstrated have an increase between 1990 and 2019 (all *P* < 0.05), but Armenia and Mongolia have a decrease between 2010 and 2019 (all *P* < 0.05).

**Conclusion:**

The prevalence of glaucoma continues to pose a significant burden across regions, ages, and genders in countries along the "B&R". It is imperative for the "B&R" nations to enhance health cooperation in order to collaboratively tackle the challenges associated with glaucoma.

**Supplementary Information:**

The online version contains supplementary material available at 10.1186/s12886-024-03464-z.

## Background

In 2013, the Chinese government introduced the visionary Belt and Road (B&R) Initiative, aiming to foster global trade, lead infrastructure development, and nurture commercial partnerships across 66 nations in Asia, Africa, South America, and Europe [1, 2]. This transformative project concentrates on establishing economic corridors, promoting connectivity, and strengthening mutual cooperation for shared prosperity and sustainable development. While the primary emphasis of the B&R Initiative (BRI) lies in economic growth and infrastructure investment, its increasingly evident influence on global health is emerging [3].

In 2017, the Chinese government further bolstered global health collaboration through the launch of the "Health Silk Road" (HSR) initiative [4]. Within the context of the Health Systems Research framework, a multitude of regional and trans-regional initiatives have been executed, encompassing the training of healthcare practitioners, the establishment of disease control centers, and the formation of research and knowledge-sharing networks. Leveraging the HSR, China has the capacity to utilize the transportation networks of the B&R Initiative to extend medical and healthcare aid to collaborating nations [5]. In light of the COVID-19 pandemic, the B&R Initiative serves as a significant platform for member countries to engage in discourse regarding clinical treatment protocols and strategies for epidemic control [6].

At present, member states face the threat of glaucoma to varying degrees. Glaucoma, a debilitating ailment primarily affecting the elderly [7], ranks as the second most prevalent cause of blindness and the foremost contributor to global irreversible visual impairment [8]. A recent investigation revealed that 3.5% of the global populace, equivalent to 64.3 million individuals, suffer from glaucoma. Of this population, approximately 5.7 million individuals experience visual impairment, while 3.1 million are blind [7, 8]. Furthermore, projections indicate that the number of individuals afflicted with glaucoma worldwide may escalate to 111.8 million by 2040 [8]. Unlike cataracts and refraction/accommodation disorders, glaucoma currently lacks a cure, leading to permanent visual impairment. Currently, member states are confronted with varying degrees of the glaucoma threat [9]. To ensure the success of the initiative, it is crucial to establish an internally consistent and comparable evaluation of glaucoma’s burden, and their trends in both China and its partner countries. The utilization of the Global Burden of Disease (GBD) 2019 framework, which incorporates a wide range of data sources and statistical modeling techniques, facilitates the comparable assessment of glaucoma burden in terms of prevalence and years lived with disability (YLDs). GBD is a comprehensive initiative spearheaded by the Institute for Health Metrics and Evaluation (IHME). It systematically collects and synthesizes global health data, utilizing advanced statistical models to provide a detailed understanding of the prevalence and impact of various health conditions, including glaucoma. This framework plays a crucial role in quantifying the overall health challenges faced by populations worldwide, informing evidence-based decision-making in public health policies and interventions. At present, there is limited knowledge regarding the prevalence and achievement of glaucoma across 66 countries participating in the B&R countries [9, 10]. This study seeks to examine and compare the burden of glaucoma between 1990 and 2019. Additionally, we aim to investigate the evolving trends of YLDs in relation to gender, age, and risk factors within the member nations of the "B&R". The findings of this research endeavor will serve as a foundation for the development of effective policies aimed at preventing and controlling glaucoma, thereby promoting the overall health of the "B&R" region.

## Methods

### Data sources and definitions

The data utilized in this investigation were obtained from the GBD 2019 study, which encompassed an estimation of 369 diseases and injuries, 87 risk factors, and various combinations of risk factors across 204 countries and territories. A comprehensive description of the methodology employed in this study has been previously published [11, 12]. Standard epidemiological measures, including prevalence and summary measures of health such as YLDs, were adopted to assess the impact of glaucoma. Age-standardized rates (ASRs) for prevalence and YLDs were calculated utilizing a global age structure from 2019. YLDs serve as a metric for quantifying the amount of time individuals lose due to diseases and injuries that diminish their health without resulting in death. The ASRs were adjusted utilizing the direct method and the world standard population to accommodate variations in the age composition of the populace. In order to guarantee transparency and the ability to reproduce our findings, our study adheres to the Guidelines for Accurate and Transparent Health Estimates Reporting (GATHER) (Table S1) [13]. All data utilized in this study were sourced from the Institute for Health Metrics and Evaluation (IHME) website.

### Statistical analyses

The prevalence and YLDs for glaucoma were determined in "B&R" countries, both in absolute numbers and ASRs. The 95% uncertainty interval (UI) was provided for each estimated metric. The 95% UI was derived by sampling 1000 draws from the posterior distribution of each quantity and identifying the 2.5th and 97.5th ordered draws of the uncertainty distribution. The ASRs for prevalence and YLDs were calculated using a global age structure from 2019, enabling comparisons across different time periods, countries, and subregions. We divided the age into nine groups: 45 to 49 years, 50 to 54 years, 55 to 59 years, 60 to 64 years, 65 to 69 years, 70 to 74 years, 75 to 79 years, 80 to 85 years, and ≥ 85 years. The study assessed the temporal patterns of disease burden between 1990 and 2019 by employing the average annual percent change values(AAPCs) through the utilization of joinpoint regression software. Additionally, the model identified 95% confidence intervals (CIs) for each segment of the trend. Moreover, we calculated the AAPCs of ASRs for YLDs related to glaucoma, categorized by gender, age, and high fasting plasma glucose levels. Furthermore, we compared the variations in AAPCs for glaucoma burden over the past decade and throughout the entire study period (1990–2019 and 2010–2019). If both the estimated AAPCs and the lower limit of the 95% uncertainty interval (UI) were positive, there was an observed increasing trend in ASRs of prevalence and YLDs. Conversely, if both the AAPCs estimate and the upper limit of the 95% UI were negative, there was an observed decreasing trend in ASRs of prevalence and YLDs (with a cut-off point of 1.5% and a larger decrease defined as ≥ 1.5%). In all other scenarios, the ASRs was considered stable. The analysis was performed using the Joinpoint Regression Program (Version 4.9.0.0. March 2021) [14]. A significance level of *P* < 0.05 was used to determine statistical significance.

## Results

Absolute number of mortality and YLDs in 1990 and 2019 caused by glaucoma attributed to modifiable dietary risk factors in each member country of the "B&R" are shown in Table 1. We found geographical differences in prevalence and YLDs of glaucoma among member countries, with China (1,338,209.52, 95% UI: 1,111,297.55–1,592,225.46; 112,528.2942, 95% UI: 77,532.87–160,898.46), India (1,297,391.73, 95% UI: 1,071,982.08–1549274.88; 120,634.0813, 95% UI: 82,875.91–167,884.62), and Indonesia (183,723.67, 95% UI: 151,064.45–219,098.40; 22,929.84187, 95% UI: 14,980.50–33050.51) being the three highest countries in 2019. The country with the lowest number of prevalence and YLDs is the Maldives in Southeast Asia (209.76, 95% UI: 169.52–254.07; 18.27972415, 95% UI: 11.96 to 26.12) in 2019.

**Table 1:** The absolute number of prevalence and YLDs due to glaucoma of B&R countries in 2019

	**Prevanlence**		**YLDs**	
**Countries**	**Number**	***95%UI***	**Number**	***95%UI***
Global	7,473,399.79	6,347,182.54 to 8,769,519.94	748,307.7133	515,635.65 to 1,044,667.15
**SDI level**				
High SDI	1,084,524.37	916,780.41 to 1,273,891.87	112,534.0245	78,104.93 to 154,478.24
High-middle SDI	1,641,067.35	1,387,922.30 to 1,922,561.74	164,966.7355	113,868.59 to 227,607.15
Middle SDI	2,525,627.89	2,125,515.19 to 2,988,280.25	246,219.3199	169,632.56 to 347,587.04
Low-middle SDI	1,449,916.97	1,210,185.57 to 1,717,083.17	138,558.6102	95,424.58 to 194,860.61
Low SDI	767,565.61	646,729.91 to 904,910.47	85,508.61347	58,215.82 to 121,783.52
**East Asia**				
China	1,338,209.52	1,111,297.55 to 1,592,225.46	112,528.2942	77,532.87 to 160,898.46
**Central Asia**				
Armenia	3979.35	3224.24 to 4792.51	421.5585509	277.53 to 601.28
Azerbaijan	7395.22	6044.15 to 8933.77	807.5446915	524.52 to 1181.62
Georgia	6553.28	5335.72 to 7943.18	708.3913687	470.46 to 1012.61
Kazakhstan	14,116.87	11,565.75 to 17,102.46	1478.639081	985.94 to 2115.74
Kyrgyzstan	4578.44	3787.32 to 5440.91	523.4107737	339.57 to 748.57
Mongolia	2185.80	1774.71 to 2633.98	261.5663943	170.20 to 378.91
Tajikistan	4211.51	3417.11 to 5119.02	493.9767556	316.56 to 728.34
Turkmenistan	2369.69	1939.59 to 2847.83	261.009947	170.69 to 372.85
Uzbekistan	14,414.17	11,548.14 to 17,780.29	1582.502745	1014.74 to 2313.09
**South Asia**				
Bangladesh	101,125.22	83,443.18 to 121,571.18	10,358.30648	6787.86 to 14,695.12
Bhutan	333.55	274.79 to 405.67	29.84674337	20.12 to 41.73
India	1,297,391.73	1,071,982.08 to 1,549,274.88	120,634.0813	82,875.91 to 167,884.62
Maldives	209.76	169.52 to 254.07	18.27972415	11.96 to 26.12
Nepal	15,633.75	13,509.40 to 17,880.70	1432.779668	977.59 to 2033.88
Pakistan	106,700.88	88,071.50 to 126,046.62	9358.402846	6344.68 to 13,254.44
**Southeast Asia**				
Cambodia	8046.26	6560.99 to 9759.45	885.1481027	579.14 to 1289.38
Indonesia	183,723.67	151,064.45 to 219,098.40	22,929.84187	14,980.50 to 33,050.51
Lao	2779.15	2245.81 to 3358.44	240.2745611	160.39 to 341.10
Malaysia	15,082.11	12,249.47 to 18,423.17	1351.884785	897.17 to 1944.79
Burma	36,319.19	28,953.38 to 45,001.56	4355.234768	2789.12 to 6453.93
Philippines	60,511.50	50,909.38 to 71,427.22	6131.574679	4212.32 to 8736.08
Sri Lanka	18,638.53	14,877.78 to 22,860.50	1471.662121	964.88 to 2112.61
Thailand	67,151.66	54,976.95 to 81,616.42	4551.913527	3123.99 to 6419.22
Viet Nam	61,181.16	51,729.09 to 71,352.27	5426.473225	3696.58 to 7565.75
**High-income Asia pacific**				
Brunei Darussalam	111.41	90.42 to 135.26	12.29524088	8.11 to 18.01
Singapore	5140.82	4306.52 to 6152.32	544.4982192	366.67 to 771.77
**North Africa and Middle East**				
Afghanistan	22,751.07	18,337.80 to 27,372.74	2975.459071	1958.43 to 4296.38
Bahrain	1107.38	882.79 to 1353.69	114.2428934	74.95 to 164.47
Egypt	102,215.24	82,664.85 to 124,638.92	11,067.67001	7265.52 to 15,896.63
Iran	181,879.99	155,555.98 to 211,333.82	22,099.85413	15,041.48 to 30,935.24
Iraq	36,404.58	29,637.32 to 44,038.82	4112.458357	2719.89 to 5962.08
Jordan	7879.24	6440.84 to 9573.20	859.0955954	570.54 to 1255.34
Kuwait	3074.77	2517.21 to 3704.21	325.7471695	215.98 to 459.83
Lebanon	8715.61	7072.30 to 10,616.33	864.9381246	576.21 to 1224.34
Oman	2534.67	2031.42 to 3080.28	246.0814766	161.78 to 352.60
Palestine	2474.88	1995.17 to 3011.93	278.6286624	179.77 to 406.42
Qatar	1246.27	1032.39 to 1489.86	149.2095133	99.92 to 210.59
Saudi Arabia	28,888.68	23,375.00 to 34,544.18	3225.660009	2107.48 to 4648.64
Syrian Arab Republic	21,467.24	17,407.58 to 26,071.63	2449.235064	1611.95 to 3574.44
Turkey	107,762.14	87,277.76 to 129,918.70	11,676.37256	7723.82 to 16,848.22
United Arab Emirates	2993.20	2359.84 to 3742.36	325.4976394	207.59 to 476.44
Yemen	19,273.78553	15,558.56 to 23,612.35	2353.561387	1525.98 to 3487.15
**Central Europe**				
Albania	1827.51	1488.06 to 2214.38	175.8650531	116.75 to 252.43
Bosnia and Herzegovina	2406.87	1956.88 to 2920.23	224.9864853	149.47 to 319.62
Bulgaria	5090.96	4042.09 to 6280.88	451.6914781	295.57 to 642.46
Croatia	3519.55	2840.99 to 4305.82	307.4423272	203.41 to 441.12
Czechia	8160.02	6594.46 to 9957.80	704.2018281	472.53 to 995.58
Hungary	9552.37	7825.04 to 11,576.32	934.7361516	615.99 to 1340.65
Montenegro	368.58	296.75 to 446.78	33.30636741	21.68 to 47.77
Macedonia	1155.05	925.08 to 1415.67	106.839011	69.76 to 156.46
Poland	28,151.54	23,761.46 to 33,080.25	2518.145902	1721.63 to 3441.09
Romania	16,318.54	13,214.05 to 19,888.60	1548.691203	1029.93 to 2198.82
Serbia	6341.47	5096.90 to 7753.75	581.1478256	379.17 to 841.44
Slovakia	3517.10	2861.20 to 4338.52	315.5117895	211.18 to 449.35
Slovenia	1739.27	1398.01 to 2126.03	149.9960642	99.51 to 214.43
**Eastern Europe**				
Belarus	8551.31	6976.65 to 10,443.72	795.5879013	532.29 to 1125.44
Estonia	989.23	796.45 to 1218.76	88.58499243	58.44 to 125.66
Latvia	2312.19	1871.03 to 2865.14	213.4766043	140.45 to 305.08
Lithuania	3356.15	2700.15 to 4118.12	311.0442683	203.94 to 446.22
Moldova	3585.58	2975.05 to 4279.80	334.4649694	226.35 to 463.53
Russian Federation	169,403.81	143,872.76 to 198,597.54	16,348.30839	11,198.98 to 22,489.08
Ukraine	43,888.12	36,720.97 to 51,940.86	4185.672623	2810.27 to 5817.71
**Western Europe**				
Cyprus	927.80	749.65 to 1124.13	104.9716307	70.15 to 150.30
Greece	14,902.37	11,936.43 to 18,266.88	1745.46265	1158.91 to 2504.26
Israel	6823.31	5578.67 to 8165.12	792.5247161	529.64 to 1119.65

**Fig. 1:**
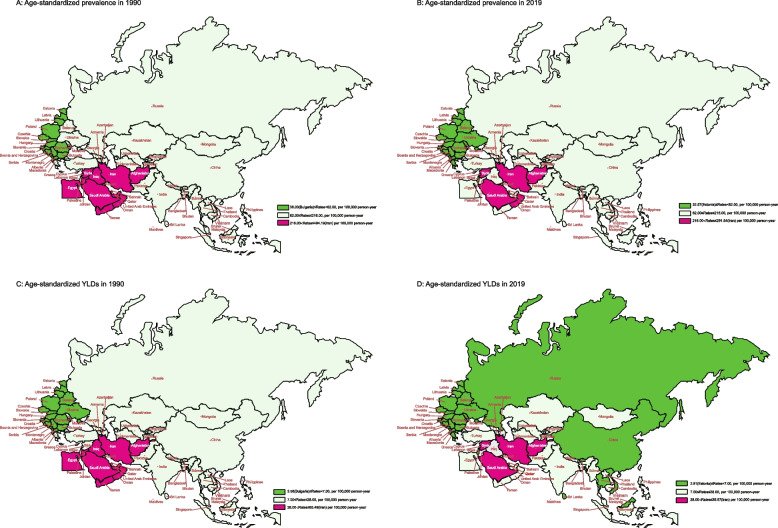
The age-standardized prevalence and YLDs due to glaucoma in 1990 and 2019 for B&R countries. **A** Age-standardized prevalence rate in 1990; **B** Age-standardized prevalence rate in 2019; **C** Age-standardized YLDs rate in 1990; **D** Age-standardized YLDs rate in 2019

Figure 1 intuitively showed the ASRs of prevalence and YLDs due to glaucoma in 1990 and 2019 in member countries of the B&R initiative. In 1990 and 2019, Eastern Europe was the region with the lowest age-standardized glaucoma prevalence and YLDs rates along the B&R, while Central Asia was the highest. On an individual country basis in 2019, Iran (291.64 per 100,000; 35.57 per 100,000), located in Central Asia, has the highest age-standardized prevalence and YLDs for glaucoma and Estonia (32.57 per 100,000; 2.91 per 100,000), located in Eastern Europe, has the lowest. See supplementary Table S2 for more details.

**Fig. 2:**
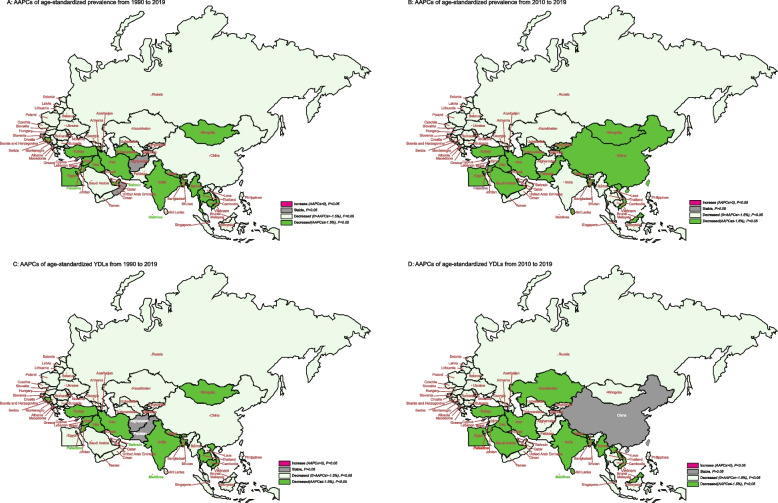
The trends of age-standardized prevalence and YLDs due to glaucoma for B&R countries. **A**The AAPCs of age-standardized prevalence rate from 1990–2019; **B** The AAPCs of age-standardized prevalence rate from 2010–2019; **C** The AAPCs of age-standardized YLDs rate from 1990–2019; **D** The AAPCs of age-standardized YLDs rate from 2100–2019

The AAPCs of ASRs of prevalence and YLDs due to glaucoma from 1990 to 2019 for the B&R countries were displayed in Fig. 2. From 1990 to 2019 and 2010 to 2019, the AAPCs of age-standardized prevalence and YLDs for glaucoma in most member countries of the B&R initiative were decreased (all *P* < 0.05). However, there are some exceptions, such as Pakistan and Afghanistan, the AAPCs of age-standardized YLDs due to glaucoma statistically increased from 1990 to 2019. In addition, there was also no decrease in AAPCs for age-standardized prevalence in Yemen and Afghanistan. Furthermore, AAPCs for glaucoma prevalence in China have decreased statistically from 2010 to 2019, but that for glaucoma YLDs have remained stable. See supplementary Table S3 for more details.

**Fig. 3:**
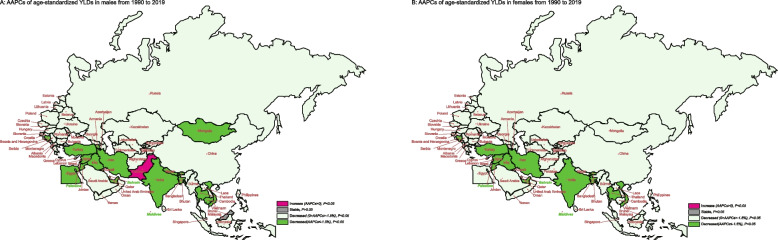
The trends of age-standardized YLDs due to glaucoma stratified by gender for B&R countries. **A** The AAPCs of age-standardized YLDs rate in male from 1990 to 2019; **B** The AAPCs of age-standardized YLDs rate in female from 1990 to 2019

Figure 3 illustrated the AAPCs of age-standardized YLDs in each member country of the “B&R” in males and females. Most of countries in the “B&R” countries showed a downward trend in AAPCs in both male and female(all *P* < 0.05), except for Pakistan (0.35, 95% UI: 0.19–0.5, *P* < 0.001)and Malaysia (-0.17, 95% UI: -0.45–0.11, *P* > 0.05). It also revealed that the trend of the age-adjusted YLDs due to glaucoma was similar in male and female in most B&R countries from 1990 to 2019. See supplementary Table S4 for more details.

**Fig. 4:**
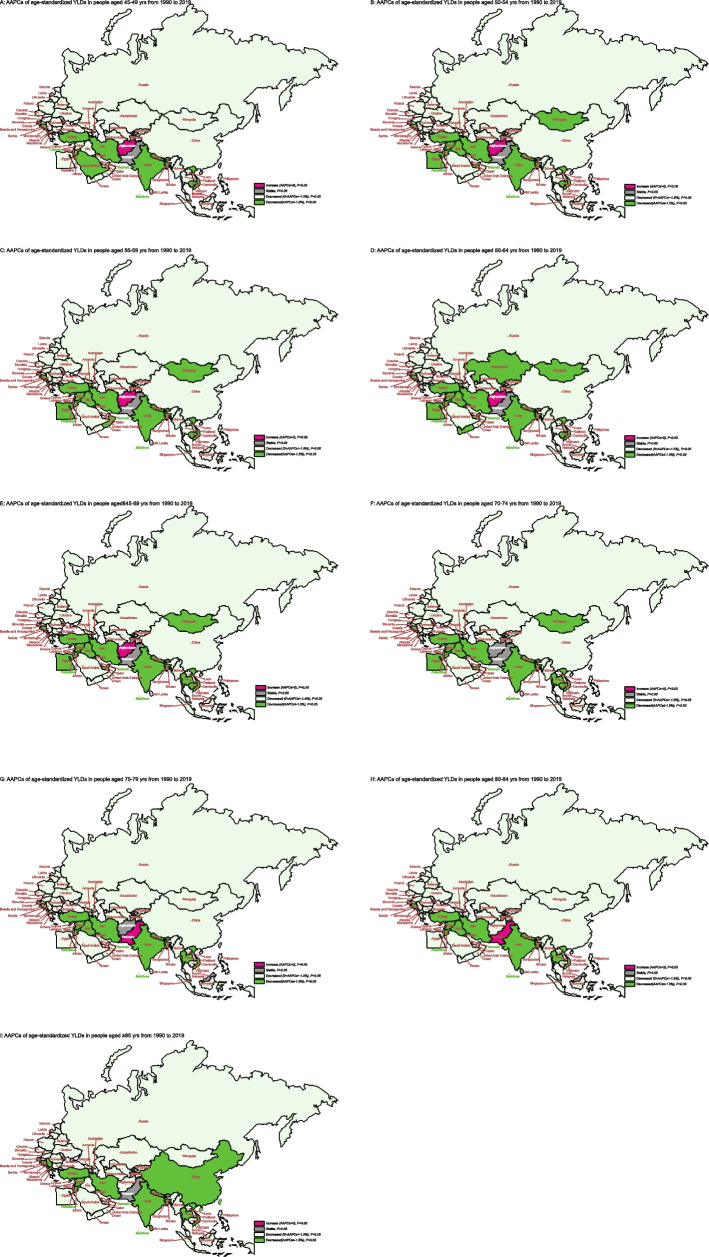
The trends of age-standardized YLDs due to glaucoma stratified by age for B&R countries. **A**The AAPCs of age-standardized YLDs rate in people aged 45–49 yrs from 1990 to 2019; **B** YLDs rate in people aged 50–54 yrs from 1990 to 2019; **C** YLDs rate in people aged 55–59 yrs from 1990 to 2019; **D** YLDs rate in people aged 60–64 yrs from 1990 to 2019; **E** YLDs rate in people aged 65–69 yrs from 1990 to 2019; **F** YLDs rate in people aged 70–74 yrs from 1990 to 2019; **G** YLDs rate in people aged 75–79 yrs from 1990 to 2019; **H** YLDs rate in people aged 80–84 yrs from 1990 to 2019; **I** YLDs rate in people aged ≥ 85 yrs from 1990 to 2019

Figure 4 showed the long-term trends of age-standardized YLDs due to glaucoma, stratified by age for 1990–2019 for the “B&R” countries. It is revealed that the age-standardized YLDs caused by glaucoma showed a decreasing trend with the increase of age in the most of B&R countries(all *P* < 0.05), except for Pakistan(45–79 years and > 85 years), Malaysia(75–84 years),Brunei Darussalam(45–49 years),Afghanistan(70–79 years). See supplementary Table S5 for more details.

**Fig. 5:**
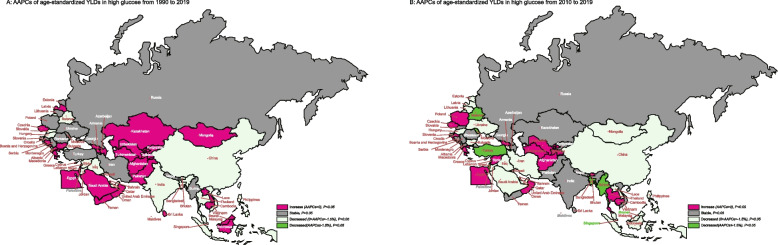
The trends of age-standardized YLDs due to glaucoma stratified by plasma glucose for B&R countries. **A** The AAPCs of age-standardized YLDs rate by high fasting plasma glucose from 1990–2019; **B** The AAPCs of age-standardized YLDs rate by high fasting plasma glucose from 2010–2019

Figure 5 illustrated the trends in age-adjusted DALYs rates for glaucoma due to high fasting glucose in B&R countries. In all Central Asian countries, the age-standardized YLDs due to glaucoma caused by fasting hyperglycemia demonstrated have a increase between 1990 and 2019(all *P* < 0.05), but Armenia and Mongolia have a decrease between 2010 and 2019 (all *P* < 0.05).

In addition, a stable or even increasing trend was observed in age-standardized glaucoma YLDs resulting from fasting hyperglycemia in most of Eastern Europe countries from 1990 to 2019. Conversely, a declining trend has been observed in the majority of nations situated in East, South, and Southeast Asia (all *P* < 0.05). See supplementary Table S6 for more details.

## Discussion

In our study, we deliberately selected the BRI countries due to their diverse populations and cultures, spanning Asia, Europe, and Africa. This diversity allows for an in-depth analysis of societal behaviors and structures, essential for our research. The BRI's unique transnational interconnectedness, facilitating cross-border flows, provides a distinct context for examining the dynamics of our study variables. Additionally, these countries are undergoing significant economic and infrastructural changes, presenting an ideal setting to observe the interplay between development and societal shifts. This concentrated approach, in contrast to a global scope, enables a focused and in-depth exploration, ensuring clarity and specificity in our findings, and avoiding the potential complexities and generalized results that a broader, global study might produce.

The current investigation involved an examination of the burden and its trends of glaucoma across a span of 30 years, commencing from 1990 to 2019, within the constituent states of the "B&R". Our findings suggest that the risk of glaucoma varied significantly among member states, particularly in the Asian region where China, India, and Indonesia exhibited the highest prevalence numbers of glaucoma in 2019. Similarly, the results of this study indicated that these three countries also had the highest numbers of YLDs due to glaucoma among the "B&R" nations in 2019. The elevated prevalence and YLDs numbers of glaucoma in China, India, and Indonesia can potentially be attributed to their status as the three most populous nations in the region [15], accounting for 31.8%, 31.5%, and 6.2% of the population, respectively.

Upon adjusting for population and age structure, our analysis revealed that the European region exhibited the lowest age-standardized prevalence and YLDs rates in both 1990 and 2019, while the Middle East demonstrated the highest rates. Previous studies have demonstrated a correlation between regions characterized by low income and low Socio-demographic Index (SDI) and an increased prevalence of glaucoma [16–19]. It is widely recognized that Europe exhibits substantial economic and social progress, accompanied by a high income, in stark contrast to the Middle East, where political instability, ongoing conflicts, terrorism, and social unrest persist [20]. Consequently, this disparity may explain the relatively lower incidence of glaucoma in Europe, juxtaposed with the disproportionately higher burden of the disease in the Middle East.

The findings of this study also indicated that a significant proportion of nations participating in the B&R initiative experienced a decline in AAPCs of age-standardized prevalence and YLDs for glaucoma from 1990 to 2019 and 2010 to 2019. This decline can potentially be attributed to the contributions of advancements in glaucoma screening and diagnostic technologies, such as automated perimetry and optical coherence tomography [21], which were developed in 1990 and first reported as novel tools for glaucoma diagnosis in 1995 [22]. Additionally, the decrease in prevalence and YLDs may also be linked to the progress made in pharmaceuticals, laser therapy, and surgical interventions aimed at managing intraocular pressure. However, it should be noted that in certain countries, namely Afghanistan and Pakistan in Central Asia, there has been no significant decrease in the AAPCs of age-standardized YLDs from 1990 to 2019. This lack of improvement can potentially be attributed to the frequent occurrence of terrorist events in these two nations [23], which have hindered the advancement of healthcare over the past two decades.

In a paradoxical manner, the AAPCs of age-standardized prevalence in China have exhibited a decline over the previous decade, while the AAPCs of age-standardized YLDs have remained constant. The former trend can potentially be attributed to a decrease in angle-closure glaucoma cases, which may be a consequence of the increased rate of cataract surgeries facilitated by the promotion of cataract rehabilitation programs in China during the past ten years. Conversely, the latter trend may be linked to a substantial rate of underdiagnosis of glaucoma within the Chinese population, resulting in patients missing the optimal window for disease management. The high underdiagnosis rate of glaucoma in China can be attributed to several factors. Firstly, the large population base, coupled with a low awareness of glaucoma, contributes to the problem. Secondly, there is a shortage of glaucoma doctors in hospitals [24], leading to varying levels of disease diagnosis and ultimately resulting in a low detection rate of glaucoma patients. Finally, the prevalence and progression of myopia among Chinese children and adolescents have been significantly high in recent years [25, 26]. Notably, Mild, moderate, and high myopia are recognized as risk factors for primary open angle glaucoma (POAG) [27], leading to an elevated incidence of glaucoma. Nevertheless, myopic patients, particularly those with high myopia, frequently exhibit atypical early signs and symptoms of POAG, which increases their vulnerability to misdiagnosis. Hence, it is imperative for China to enhance the public's awareness regarding glaucoma, improve the diagnostic proficiency of glaucoma specialists in primary healthcare facilities, and bolster efforts in myopia prevention and control.

Furthermore, we assessed the age-adjusted YLDs due to glaucoma by sex in each member country of the “B&R” in depth. Our research reveals that the trend of the age-adjusted YLDs due to glaucoma was similar in male and female in most B&R countries from 1990 to 2019. This similarity can be attributed to the adoption of a uniform glaucoma prevention strategy for both genders in these regions. However, numerous studies have consistently demonstrated that men have higher glaucoma burden than women in the majority of global regions [28]. This suggests that the implementation of gender-specific strategies for glaucoma prevention and treatment may lead to more effective outcomes in mitigating the burden associated with this condition.

In addition, our research also indicates that the age-standardized YLDs caused by glaucoma showed a decreasing trend with the increase of age in the most of B&R countries, except for Malaysia, Pakistan, and Brunei Darussalam. This trend could potentially be attributed to the increased life expectancy experienced in these nations. Overall, variations in the trend of age-standardized YLDs for glaucoma were observed among different age groups within various member countries of the "B&R" initiative. These disparities could potentially be ascribed to disparities in genetic predisposition, lifestyle preferences, dietary patterns, and localized environmental factors prevalent in diverse geographical regions [28, 29].

Diabetes has emerged as a prevalent chronic ailment on a global scale, exhibiting a substantial surge in its occurrence over the past few decades. This condition, referring to diabetes, is closely linked to a diverse range of ocular complications, encompassing retinopathy, cataracts, uveitis, and neovascularization. Furthermore, a number of investigations have indicated a potential correlation between diabetes and an elevated susceptibility to glaucoma [30]. Nevertheless, the mechanisms underlying the relationship between diabetes and glaucoma remain unclear. Speculatively, this could be partly due to the heightened incidence of neovascular glaucoma associated with high blood glucose levels. In addition, regular ocular examinations in hyperglycemic patients make it easier to detect glaucoma-like changes in the optic fundus. With this in mind, the present study endeavors to evaluate the trend of age-adjusted YLDs attributed to glaucoma resulting from high fasting glucose levels in countries associated with the B&R Initiative. During the past thirty years, our observations have indicated a consistent or potentially rising trend in age-standardized YLDs linked to glaucoma resulting from fasting hyperglycemia in a large number of Central Asian and Eastern European countries. This trend may be attributed to an augmented prevalence of diet-related type 2 diabetes, potentially caused by excessive consumption of unprocessed red meat, processed meat, and potatoes in these regions [31]. Conversely, a decreasing trend has been observed in many countries situated in East, South, and Southeast Asia. This observation aligns with the economic progress, population expansion, and increased urbanization experienced in Southeast and East Asia during the preceding three decades. Furthermore, it is noteworthy that the trend in age-standardized YLDs associated with this condition has exhibited a downward trajectory in a considerable portion of B&R countries over the past decade, as compared to the preceding three decades, indicating that these regions have made commendable strides in enhancing dietary habits and implementing effective strategies for the prevention and management of diabetes in recent years [31].

This study is subject to several limitations. Firstly, it is influenced by the limitations inherent in the GBD methodology, as previously discussed. Secondly, the measurement of visual disability for calculating YLDs may not be consistent across different demographic and sociodemographic groups. Moreover, the identification of glaucoma cases in the GBD data might not always clearly differentiate true glaucoma from conditions with similar optic disc characteristics, such as in individuals with myopia. Lastly, disparities in the quality and comprehensiveness of available data may contribute to the observed variations when comparing different 'B&R' countries.

## Conclusion

This study conducted a comparative analysis of the burden and trends of glaucoma, as well as an examination of age-standardized YLDs stratified by gender and age, within the "B&R" countries from 1990 to 2019 and 2010 to 2019. The findings indicate that the burden of glaucoma varied among member states, with China, India, and Indonesia exhibiting the highest absolute numbers. The prevalence and YLDs of glaucoma, adjusted for age, exhibited a downward trend, while the AAPCs of age-standardized YLDs varied across gender and age cohorts in countries belonging to the "B&R" initiative. Consequently, fostering health cooperation and resource sharing among member states within the framework of a community with a shared future for mankind will facilitate collective efforts in tackling the risks and challenges associated with glaucoma.

### Supplementary Information

**Supplementary Material 1. **

**Supplementary Material 2. **

**Supplementary Material 3. **

**Supplementary Material 4. **

**Supplementary Material 5. **

**Supplementary Material 6. **

## Data Availability

For accessing the data utilized in these analyses, kindly navigate to the GBD 2019 website of the Global Health Data Exchange.https://vizhub.healthdata.org/gbd-results/

## Abbreviations

ASRs: Age-standardized rates
AAPCs: Average annual percent change values
B&R: Belt and Road
CIs: Confidence intervals
DALYs: Disability adjusted life years
GATHER: Guidelines for Accurate and Transparent Health Estimates Reporting
HSR: Health Silk Road
IHME: Institute for Health Metrics and Evaluation
POAG: Primary open angle glaucoma
SDI: Socio-demographic Index
UI: Uncertainty interval
YLDs: Years lived with disability
